# Enhancing Optic Nerve Head Swelling Detection Through Deep Learning Classification of 3D OCT Images

**DOI:** 10.18502/jovr.v21.17374

**Published:** 2026-10-26

**Authors:** Seyed Kianoosh Naghibzadeh, Parviz Abdolmaleki, Mohammad Hasan Shahriari, Hadi Kamkar, Bita Shalbafan, Hajar Aminzadeh, Moslem Afrashtehmehr, Mozhgan Alipour, Mohsen Azarmina

**Affiliations:** ^1^Department of Biophysics, Faculty of Biological Sciences, Tarbiat Modares University, Tehran, Iran; ^2^Department of Health Information Technology and Management, School of Allied Medical Sciences, Shahid Beheshti University of Medical Sciences, Tehran, Iran; ^3^Labbafinejad Clinical Research Center, Shahid Beheshti University of Medical Sciences, Tehran, Iran; ^4^Department of Cognitive Modeling, Institute for Cognitive Science Studies, Tehran, Iran; ^5^Functional Neurosurgery Research Center, Shohada Tajrish Comprehensive Neurosurgical Center of Excellence, Shahid Beheshti University of Medical Sciences, Tehran, Iran; ^6^Ophthalmic Research Center, Research Institute for Ophthalmology and Vision Science, Shahid Beheshti University of Medical Sciences, Tehran, Iran

**Keywords:** 3D ResNet, Deep Learning, Machine Learning, Optic Nerve Head, Optic Nerve Swelling, Optical Coherence Tomography

## Abstract

**Purpose:**

To leverage deep learning (DL) to process optical coherence tomography (OCT) images for the detection of optic nerve head (ONH) abnormalities, particularly optic disc swelling.

**Methods:**

The study employed a dataset of 795 pre-processed OCT images, comprising 561 images with structural abnormalities and 234 images with normal ONH structures. A DL architecture, namely a 3D ResNet model, was implemented to classify the images.

**Results:**

The 3D ResNet model achieved an accuracy of 93.7%, an F1-score of 0.87, and an AUC of 0.96 on the test dataset, demonstrating high precision in distinguishing between normal and abnormal ONH structures.

**Conclusion:**

The application of 3D ResNet for classifying ONH OCT images demonstrated promising results for the automated detection of ONH swelling. This approach may support clinical decision-making by improving diagnostic accuracy and reducing reliance on manual image interpretation.

##  INTRODUCTION

Idiopathic intracranial hypertension (IIH) is a disorder characterized by elevated cerebrospinal fluid pressure within the subarachnoid space surrounding the brain and spinal cord, without an identifiable underlying cause. It affects 1 in 100,000 individuals annually and is approximately 20 times more common in young women with obesity.^[1,2]^ Common symptoms include headache, pulsatile tinnitus, and vision loss.^[3]^ Visual impairment is a significant complication of IIH because of optic nerve dysfunction.^[4,5]^ A key clinical feature of IIH is optic disc swelling, which manifests as optic disc boundary blurring and vascular obscuration.^[6]^ Traditional clinical assessments rely on qualitative evaluations, which are subject to inter-rater variability and may lead to delayed diagnosis.^[7]^ Improved biomarkers are necessary for timely and accurate diagnosis.

Optical coherence tomography (OCT) is a noninvasive, high-resolution imaging technique for visualizing optic nerve head (ONH) abnormalities, including swollen discs, as well as changes in the retinal nerve fiber layer (RNFL) and retinal pigment epithelium/Bruch's membrane (RPE/BM) associated with acute and chronic changes in intracranial pressure (ICP).^[8,9]^ While OCT has demonstrated potential for diagnosing IIH-related conditions, traditional analysis methods require expert interpretation and are time-consuming. The integration of deep learning (DL) into OCT analysis offers a promising approach to enhance diagnostic accuracy and efficiency. Specifically, DL-based analysis of OCT images may improve the diagnostic accuracy and efficiency of IIH assessment.^[10]^

**Figure 1:**
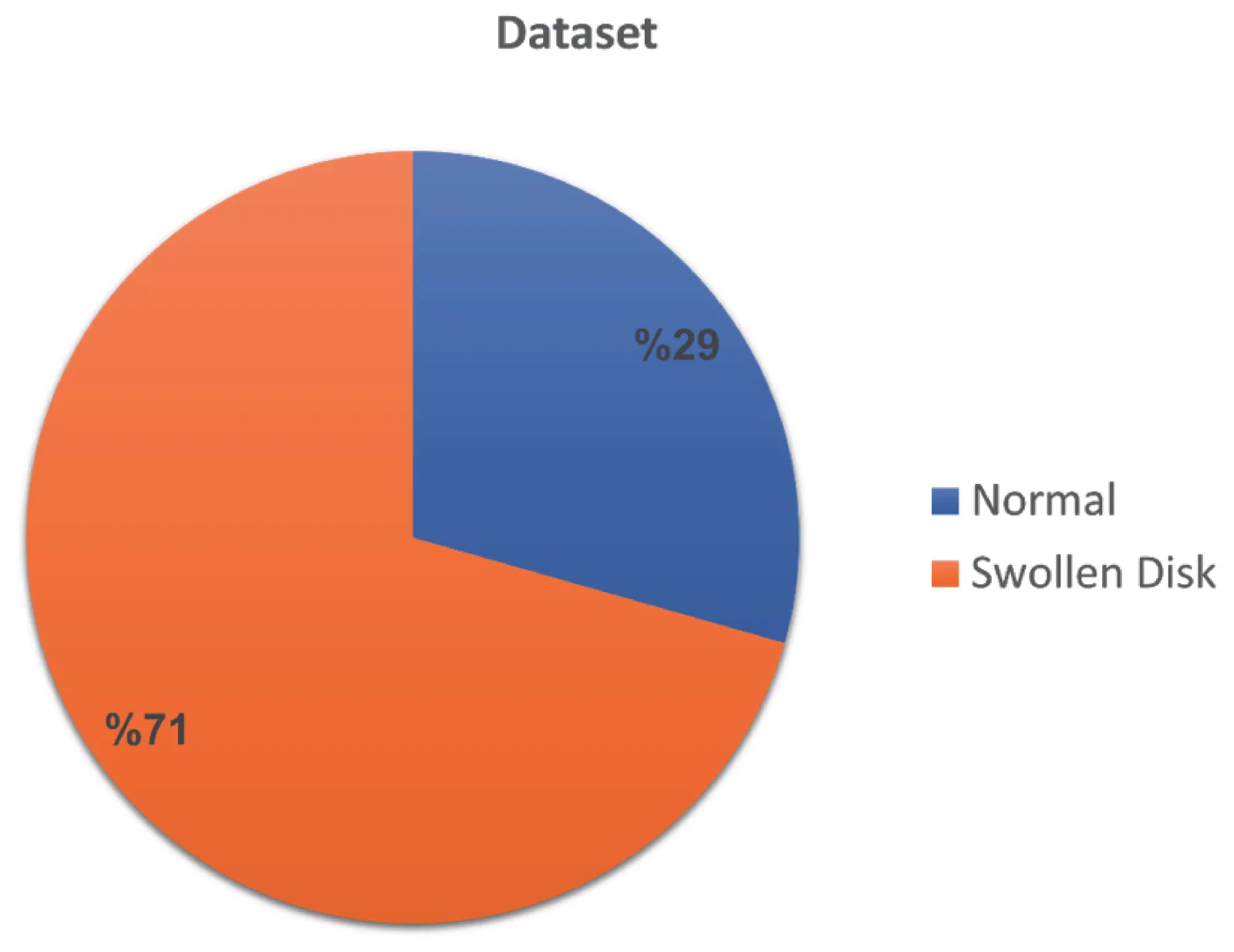
Dataset composition and class distribution of the OCT volumes (normal vs. swollen discs).

DL, particularly convolutional neural networks (CNNs), has shown success in medical image classification. CNNs are particularly useful for identifying image patterns and recognizing objects, faces, and scenes.^[11,12]^ A significant challenge in applying DL to OCT image analysis is the need for high-quality, annotated datasets, as well as the need to ensure model generalizability across different OCT devices and patient populations.^[13]^ The labor-intensive nature of manual labeling, coupled with the need for expert knowledge to accurately categorize images, can limit the scalability of DL applications in ophthalmology.^[14]^ Moreover, the generalizability of these models across different OCT devices and patient populations remains a crucial area for further investigation.^[15]^

This study aimed to address these challenges by developing a 3D ResNet model to classify OCT images of the ONH into normal or swollen disc categories. By leveraging DL techniques, this study sought to improve automated diagnostic tools, enhance clinical workflows, and support clinicians in making more efficient and accurate diagnoses.

This study hypothesized that a DL model, specifically a 3D ResNet architecture, could accurately differentiate between normal and swollen discs, improving diagnostic precision and reducing dependence on manual interpretation. The primary research question examined whether the model could achieve high accuracy and robustness across different OCT datasets, thereby overcoming current diagnostic limitations and evaluating its performance to help determine its potential for clinical integration and its role in advancing early detection and monitoring of ONH swelling.

##  METHODS

### Study Design

This was a retrospective cross-sectional diagnostic accuracy study using OCT volumes to develop and test a DL classifier for ONH swelling detection.

### Dataset

In this study, OCT images were obtained using the DRI OCT Triton, swept-source 3D OCT system (Topcon Corporation, Tokyo, Japan), with outputs provided in FDA format. The study utilized a comprehensive dataset comprising 795 OCT images, collected from two ophthalmic clinics (Labbafinejad Hospital, Tehran, Iran, and Negah Eye Hospital, Tehran, Iran). Images encompassed a wide array of conditions, focusing on the ONH with classifications into two primary categories: normal and swollen discs. Finally, we obtained a dataset comprising 561 images with swollen discs and 234 normal images to build the model [Figure 1]. The dataset's richness lies in its size, diversity, and labels, representing a broad spectrum of patient demographics and pathological variations, thereby providing a robust foundation for developing and testing the DL model. Two board-certified, fellowship-trained neuro-ophthalmologists independently labeled all OCT volumes; any disagreements were resolved by consensus in a joint review session. Additional demographic details, including gender distribution and age distribution, were included to describe dataset representativeness [Figures 2 & 3].

**Figure 2:**
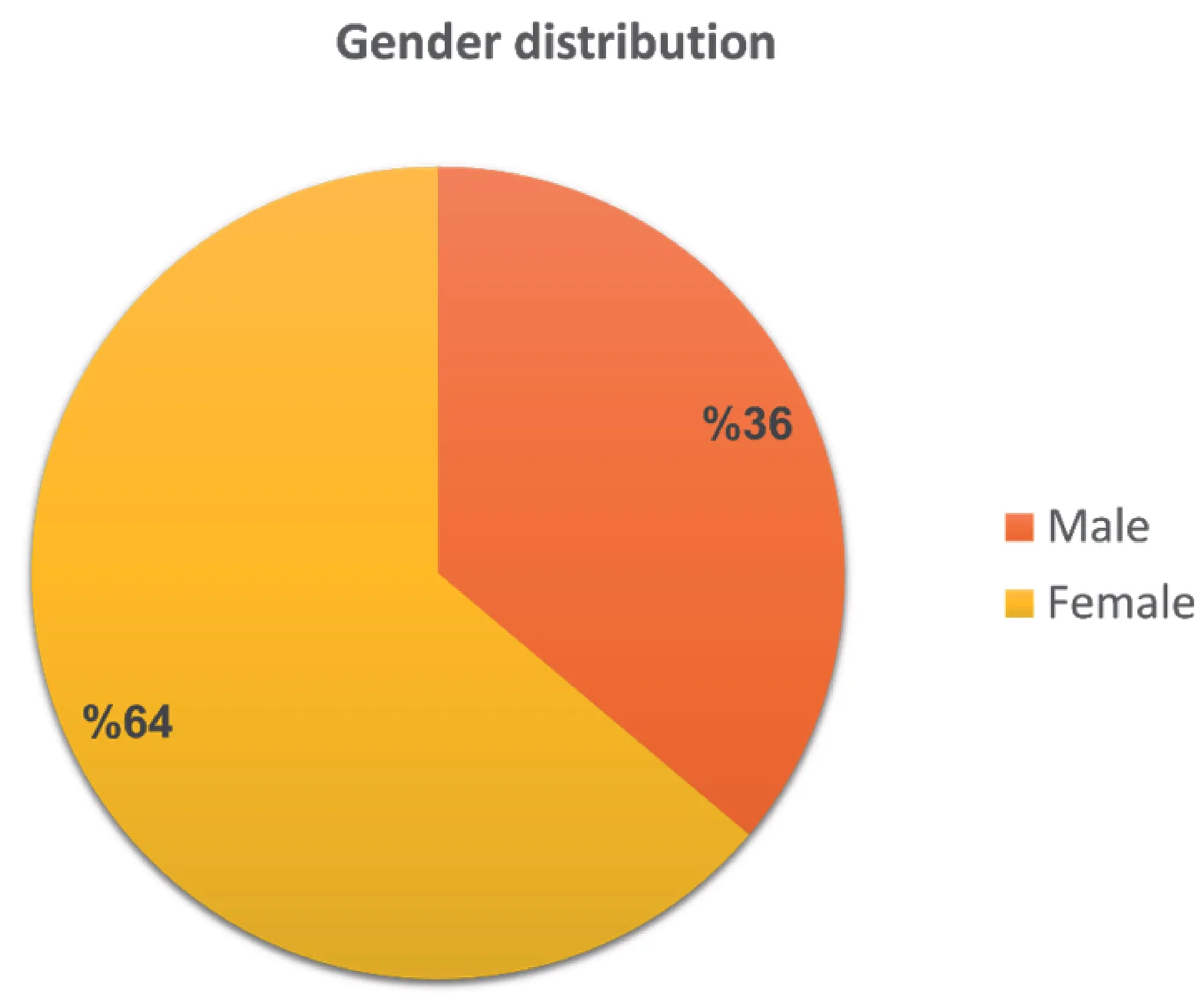
Gender distribution of participants in the OCT dataset.

**Figure 3:**
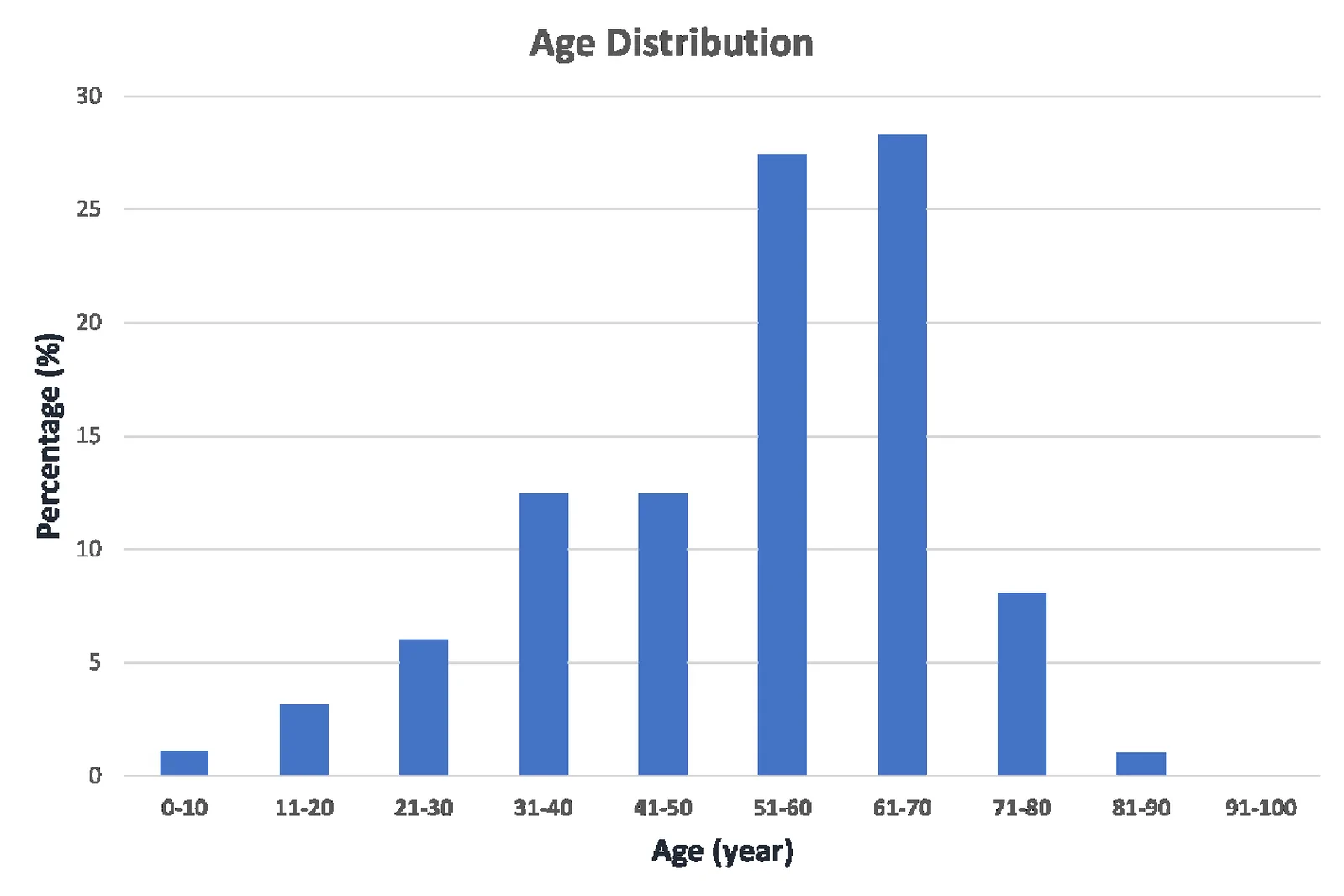
Age distribution (percentage) of participants in the OCT dataset.

### Preprocessing

Preprocessing played a pivotal role in preparing the dataset for effective model training. Before the data were entered into the model, a series of preprocessing steps were performed to improve the quality of the images. A visual representation (flowchart) of the preprocessing steps has been included for clarity [Figure 4]. The dataset underwent preprocessing, including cropping, resizing, normalization, and augmentation techniques (rotation, flipping, and scaling).

Once the images were cropped, they were resized to reduce their dimensions before being fed into the model. In the next step, data normalization was performed by dividing pixel values by 255 [Formula 1], which is one of the effective methods for normalization.^[3]^ To address the challenge of class imbalance, a combination of techniques such as rotation, flipping, and scaling was employed, enhancing the model's ability to generalize from the training data to unseen images. Furthermore, a region-of-interest (ROI) extraction was performed to focus on the ONH, thereby reducing background noise and irrelevant anatomical structures. In the last preprocessing stage, the dataset was divided into training and testing sets. Therefore, 80% of the dataset was allocated to the training set and 20% to the testing set. 


$$X_{norm}=\frac{x_{i}}{255}$$

**Formula 1.** Normalization.

**Figure 4:**
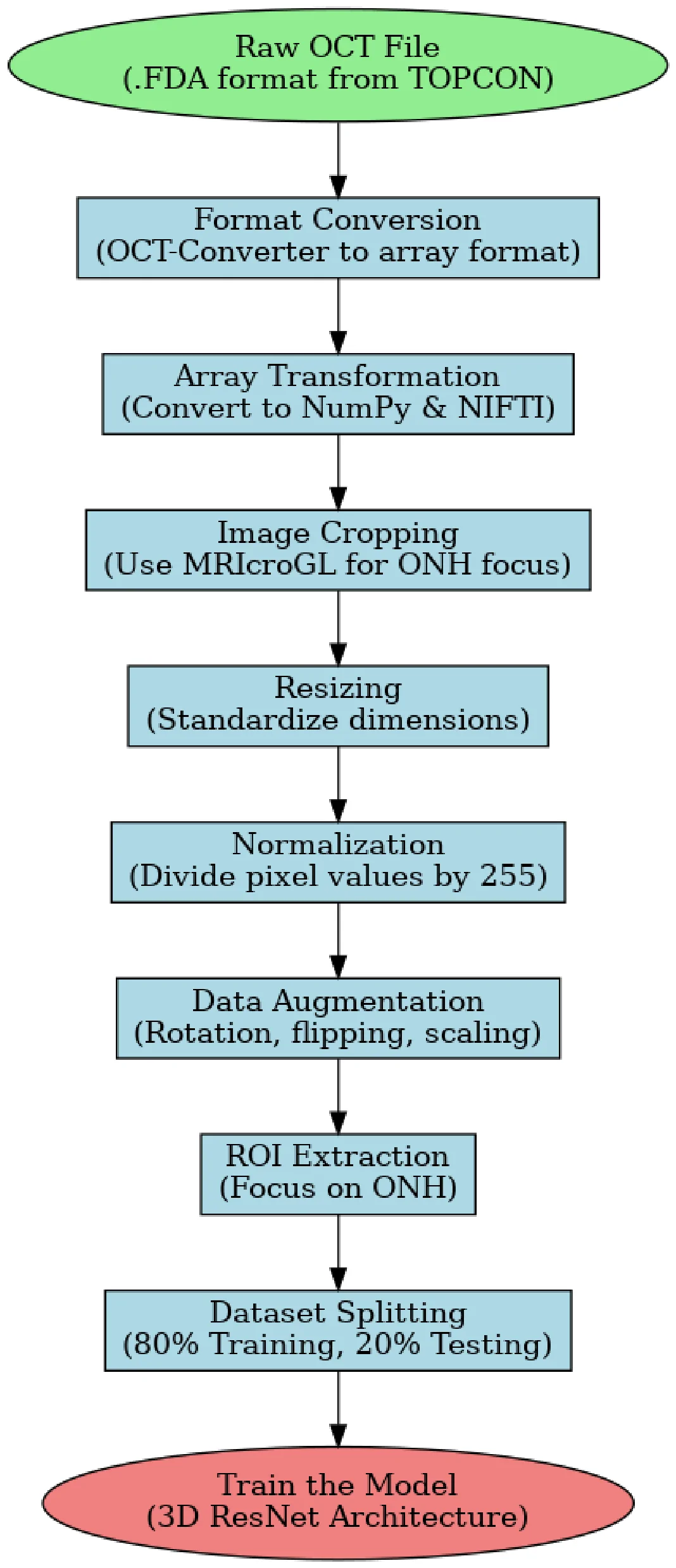
Flowchart of the preprocessing pipeline applied to the 3D OCT volumes.

**Figure 5:**
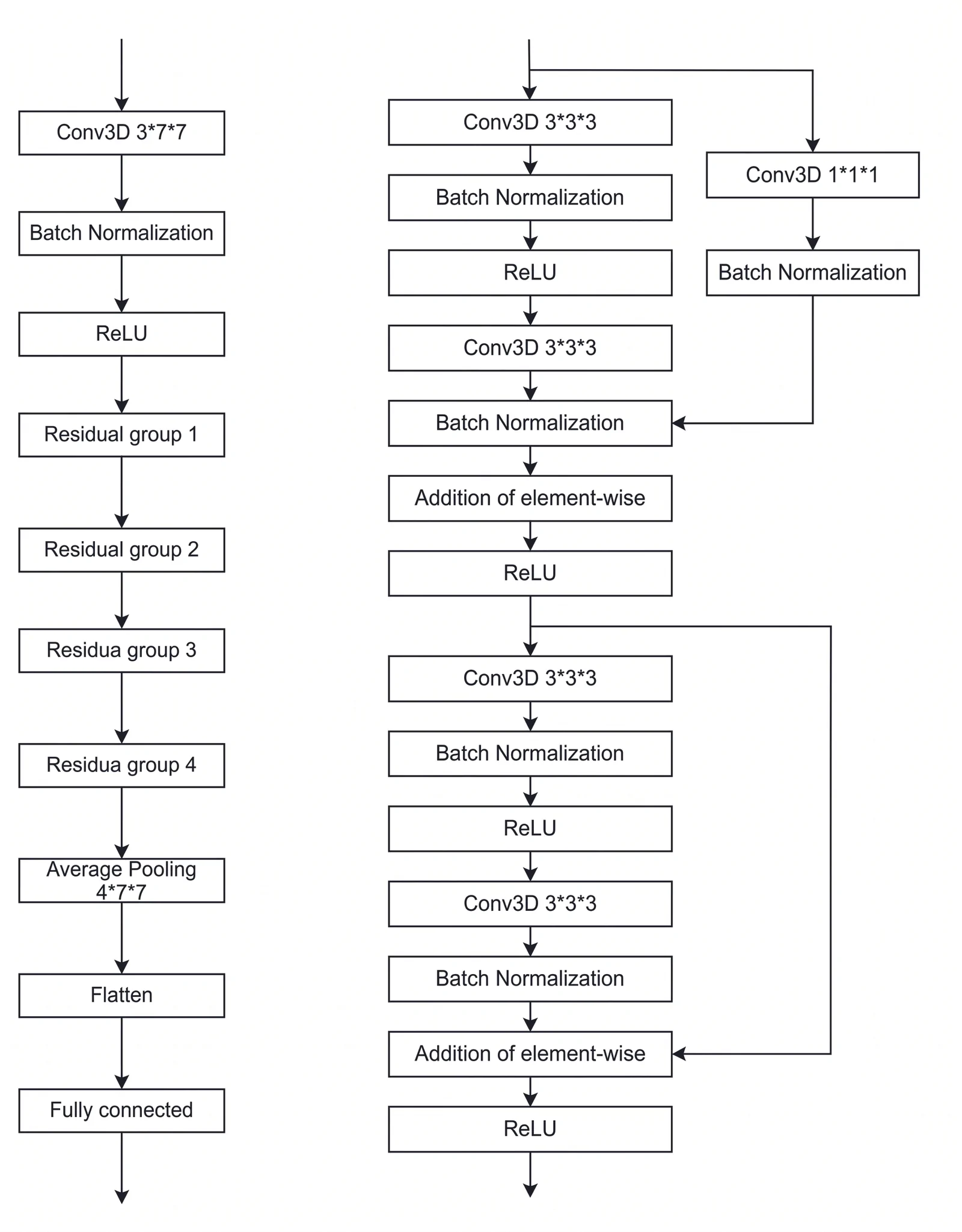
3D ResNet network architecture. (a) General structure of the 3D network with 4 residual groups. (b) Architecture of a residual group consisting of two residual blocks.

### Modeling

In recent years, CNN structures have been proposed for image classification tasks and have succeeded in ImageNet competitions.^[16]^ However, deep networks cannot be created by simply assembling several convolutional layers; these deep CNN models are capable of solving complex problems. Training a deep model is challenging because of the vanishing gradient issue. In the backpropagation training process, repeated multiplication could make the gradient infinitely small when it is backpropagated to previous layers. Therefore, the performance of deep networks may be degraded.

A 3D ResNet architecture for 3D OCT images was chosen due to its ability to handle volumetric medical imaging. Compared to other architectures, 3D ResNet provides improved feature extraction and classification. The 3D ResNet architecture is shown in Figure 5. The core idea behind ResNet is introducing a “shortcut connection” that skips some layers.^[17]^ The architecture of the pre-trained 3D-ResNet that was used in this study is shown in Figure5. This architecture includes convolutional and pooling layers as encoders to extract features from a three-dimensional image. These features are then passed through fully connected layers to produce the final output. The architecture features residual blocks with mixed complexities, where three-dimensional complexities are addressed in the early layers and two-dimensional complexities in the upper layers. Mixed convolutions are implemented using a 3 × 3 × 1 kernel followed by a 1 × 1 × 3 kernel. The residual blocks also include batch normalization and the ReLU activation function.

### Hyperparameters

The optimization of hyperparameters was critical for the model's success. The learning rate was dynamically adjusted to find the ideal balance between convergence speed and stability. The model employed a stochastic optimization optimizer, with hyperparameters fine-tuned to enhance learning efficiency. The choice of activation functions, batch sizes, and the number of epochs were all carefully calibrated based on preliminary tests and existing literature to maximize model performance.^[18]^ The model was trained over a span of 20 epochs, with a learning rate set to < 0.001. This configuration was chosen to ensure a gradual and effective learning process, minimizing the loss function efficiently while preventing the model from getting stuck in local minima.

### Training and Testing

The training process was conducted using a split of the dataset into 80% for training and 20% for testing, ensuring a comprehensive evaluation of the model's performance on unseen data. The model was trained using the Adam optimizer, a decision influenced by its adaptive learning rate capabilities, which significantly improved the convergence speed. The training process was iterated over 20 epochs, with early stopping implemented to prevent overfitting. The testing phase was crucial for assessing the model's generalization ability, executed on the held-out test set, which was not exposed to the model during the training phase.

##  RESULTS

The implementation of a 3D ResNet architecture for the classification of OCT images into normal and swollen disc categories was evaluated by measuring accuracy, sensitivity, specificity, F1-score, loss score, and the area under the receiver operating characteristic curve (AUC).

To evaluate the model's diagnostic accuracy, the following metrics were calculated: accuracy, F1-score, and loss score, together with sensitivity and specificity.^[19,20]^

Accuracy evaluates the model's total number of correct predictions across all classes, serving as a general measure of its effectiveness. The model achieved 93.7% accuracy on the test dataset. The accuracy of the training data was 99.5%.

On the test dataset, sensitivity was 92.5% and specificity was 94.9%, providing balanced performance between the detection of swollen discs and the correct identification of normal cases. The loss score quantitatively represents the model's prediction error, with lower values indicating better performance.

The model achieved F1-scores of 0.87 on the test dataset and 0.899 on the training dataset [Table 1].

Receiver operating characteristic (ROC) analysis demonstrated strong classification ability, with an AUC of 0.96 for the test data [Figure 6].

**Table 1:** 3D ResNet modeling results

**Metric**	**Test dataset**	**Training dataset**
Accuracy	93.7%	99.5%
F1-score	0.87	0.899
Loss score	0.106	0.007
Sensitivity	92.5%	–
Specificity	94.9%	–

**Figure 6:**
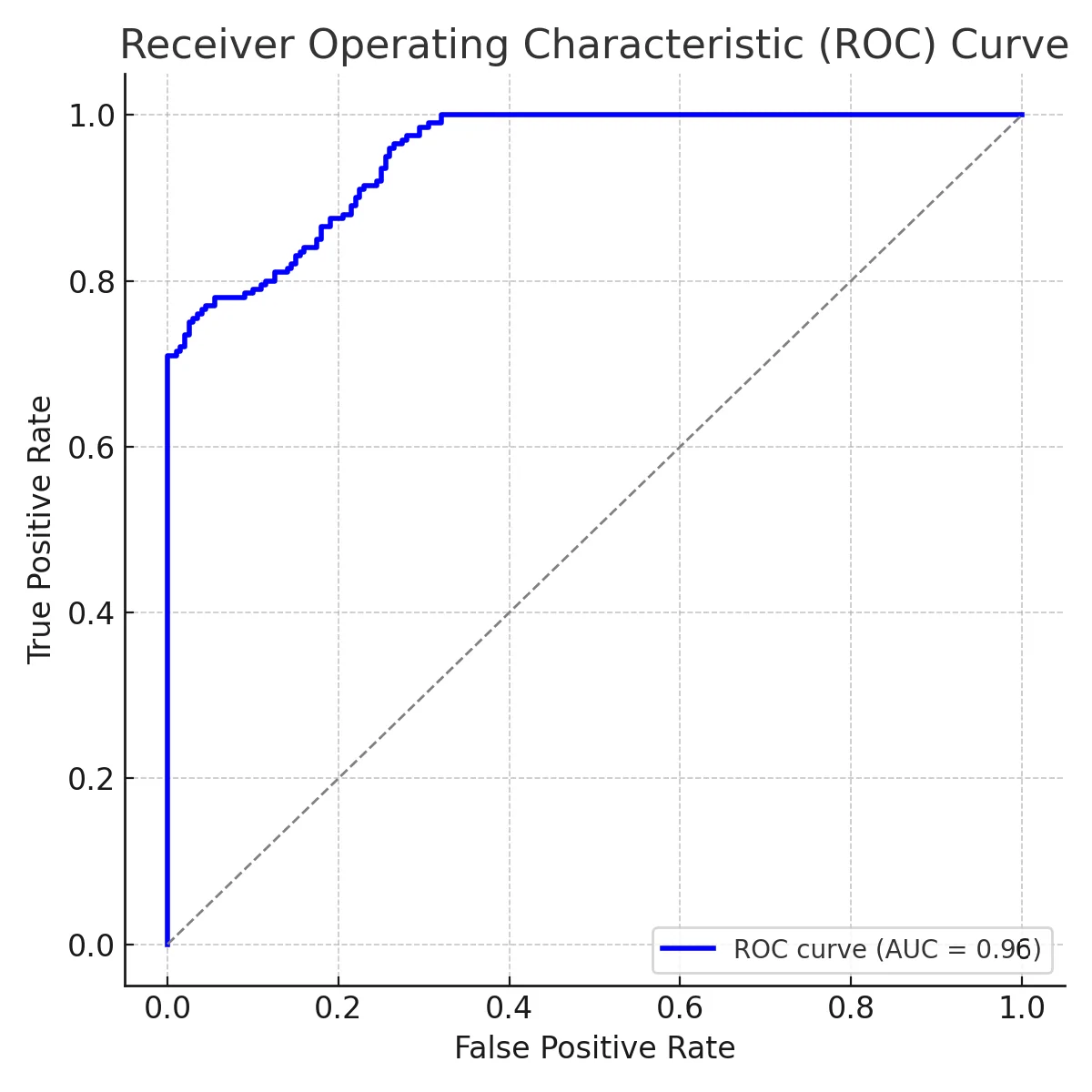
ROC curve for the 3D ResNet model on the test dataset.

##  DISCUSSION

This study developed and evaluated a 3D ResNet model for OCT-based classification of ONH abnormalities, demonstrating strong diagnostic potential. The model's high accuracy and F1-scores highlight its effectiveness in distinguishing between normal and swollen discs, reinforcing the viability of DL in medical imaging. Compared to traditional diagnostic methods that rely on subjective expert evaluation, our approach enhances consistency, reduces inter-rater variability, and offers a scalable solution for clinical implementation.

The findings align with prior research demonstrating the success of ResNet architectures in ophthalmic imaging. For instance, Jee et al used ResNet-50 to classify central serous chorioretinopathy progression, achieving 95% accuracy and an F1-score of 0.925 on a dataset of 832 images.^[21]^ While their study focused on a different condition, the comparison underscored the versatility and robustness of DL in medical image classification.

Several factors contributed to the reliability of our results, including a comprehensive dataset, rigorous preprocessing, and the use of a well-established DL model. This methodological rigor aligns with best practices recommended by Huang et al, who emphasize the importance of a systematic and robust methodology for developing AI models for medical diagnostics.^[22]^ However, challenges, particularly data imbalance and the need for extensive annotated datasets, remain. This study mitigated class imbalance through preprocessing techniques and weight coefficients, a strategy also supported by Kingma and Ba in their development of the Adam optimizer.^[23]^

The results of this study also contribute to the ongoing discourse on integrating AI into healthcare, particularly in areas where rapid, accurate diagnostics are critical. Automated OCT image analysis through DL offers a promising avenue to enhance diagnostic processes, reduce the workload for medical professionals, and potentially improve patient outcomes. As highlighted by Ran et al, the use of AI for diagnostics signals a shift toward more data-driven, precise, and efficient healthcare practices, in which technologies like the one developed in this study play a pivotal role.^[24]^ Nevertheless, the successful implementation of AI in clinical settings requires further validation, regulatory considerations, and seamless integration with existing medical infrastructure.

Despite the promising results, this study has limitations that warrant further consideration. The absence of an external test dataset restricts generalizability, as model performance has only been validated on internal data. Additionally, this study focused exclusively on normal and swollen discs. Other abnormalities, such as coloboma, glaucomatous cupping, and optic nerve hypoplasia, were not included, which may limit generalizability. Future research should focus on evaluating the model using external datasets from diverse populations and multiple OCT devices to ensure broader applicability. Additionally, investigating other DL architectures and integrating multimodal imaging data could further enhance diagnostic accuracy and clinical utility.

A significant limitation of this study is the absence of validation on an external dataset from different clinical centers. While our internal validation using 20% unseen data demonstrated promising results, external validation across diverse patient populations and imaging devices is essential to establish the model's generalizability in real-world clinical settings. Future work will prioritize multicenter validation to address this limitation.

This study contributes to the growing body of research on AI-driven medical imaging by demonstrating the feasibility of DL-based classification of ONH abnormalities. While challenges remain, continued advancements in AI have the potential to revolutionize ophthalmic diagnostics, ultimately improving patient care and clinical decision-making.^[25]^

In summary, this study developed and evaluated a 3D ResNet model for classifying OCT images, achieving 93.7% accuracy and an F1-score of 0.87. The model effectively distinguished between normal ONHs and those with optic disc swelling, facilitating timely interventions.

By extending ResNet architectures to 3D OCT images, this study demonstrated the versatility of DL in medical imaging. Despite challenges such as data imbalance, the model leverages spatial information to improve diagnostic accuracy and reliability.

Future research should use larger and more diverse datasets, integrate the model into clinical workflows, and compare it with other DL methods and traditional image analysis techniques. These findings contribute to AI-driven ophthalmic diagnostics and may advance the development of automated tools that improve patient care.

##  Financial Support and Sponsorship

None.

##  Conflicts of Interest

None.
